# Foxp2 mutations and abnormal brain and gastrointestinal development: insights from animal models of speech-language and autism spectrum disorders

**DOI:** 10.3389/fnana.2026.1783101

**Published:** 2026-03-11

**Authors:** Eriko Fujita-Jimbo, Genri Kawahara, Takashi Momoi

**Affiliations:** 1 Department of Pediatrics, Jichi Medical University, Shimotsuke-shi, Tochigi, Japan; 2 Department of Pathophysiology, Tokyo Medical University, Tokyo, Japan

**Keywords:** animal models, autism spectrum disorder (ASD), brain-gut-microbiome axis, cerebral cortex development, Forkhead-box Protein P2 (FOXP2), speech and language disorder (SLD), Wnt signaling

## Abstract

Autism spectrum disorder (ASD) and speech and language disorder (SLD) are distinct neurodevelopmental conditions, yet both share overlapping communication impairments. *Forkhead box P2* (*FOXP2*), a key transcription factor involved in speech and language development, harbors pathogenic mutations such as R553H, which cause SLD and have been suggested to contribute to aspects of ASD-related phenotypes. This review synthesizes insights from animal models to explore the molecular mechanisms by which *Foxp2* mutations disrupt the development of the cerebral cortex, thalamus, and enteric nervous system. We highlight findings from heterozygous *Foxp2* mutants and discuss severe phenotypes observed in homozygous *Foxp2* mutants (*Foxp*2^R552H/R552H^ and *Foxp*2^R552H/R552H^/mCherry-Tg mice), including profound ultrasonic vocalization deficits, brain malformations, and early lethality. Notably, these mice exhibit gastrointestinal abnormalities involving the epithelium, smooth muscle, and enteric nervous system, which are linked to impaired autoregulation and interference with Wnt signaling during development. Such observations underscore the relevance of the brain–gut–microbiome axis and Hirschsprung-like pathology in neurodevelopmental disorders. Finally, this review discusses future directions using gene-editing approaches in non-mammalian models—zebra finches, zebrafish, and *Drosophila*—to dissect neural networks underlying intellectual disability and communication deficits. Collectively, these studies provide a framework for understanding *FOXP2*-related molecular mechanisms in the pathogenesis of ASD and SLD.

## 1 Introduction

Autism spectrum disorder (ASD) is a neurodevelopmental condition characterized by deficits in communication and social interaction, along with restricted interests and repetitive behaviors. The estimated prevalence of ASD is approximately 2–5% among children (Khachadourian et al., 2023). ASD imposes a significant public health burden worldwide, with an increasing prevalence and limited effective treatments, highlighting the urgent need for mechanistic insights and novel therapeutic strategies. Common comorbidities of ASD include intellectual disability, epilepsy, language disorders, attention-deficit/hyperactivity disorder (ADHD), sleep disturbances, and gastrointestinal (GI) dysfunction (Khachadourian et al., 2023). Increasing evidence indicates that both genetic and environmental factors contribute substantially to ASD etiology. Genetic and epigenetic factors encompass chromosomal structural abnormalities such as copy number variations (CNVs), sequence variations including single-nucleotide polymorphisms (SNPs), gene mutations, and epigenetic modifications including DNA methylation and histone modifications (Masini et al., 2020; Yasuda et al., 2023).

**Figure 1:**
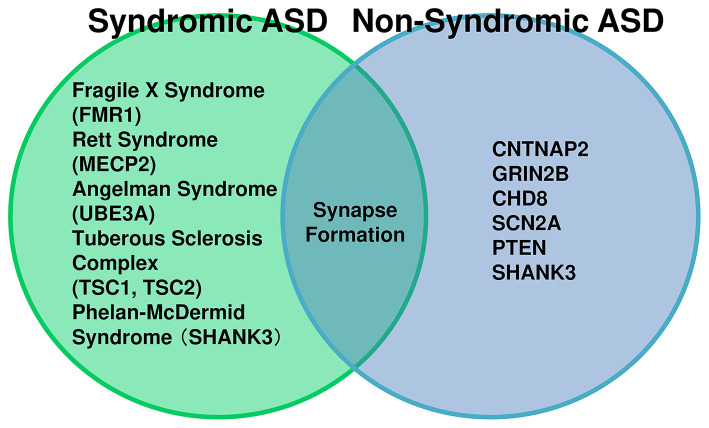
Relationship between syndromic and non-syndromic ASD and their genetic underpinnings. The **left circle** represents syndromic ASD, where autism spectrum disorder (ASD) symptoms occur as part of a broader clinical syndrome. Examples include Angelman syndrome (*UBE3A*), Rett syndrome (*MECP2*), Fragile X syndrome (*FMR1*), Phelan-McDermid syndrome (*SHANK3*), and Tuberous Sclerosis Complex (*TSC1, TSC2*). In these cases, ASD is considered a component of the syndrome. The **right circle** represents non-syndromic ASD, characterized by isolated ASD without additional syndromic features. High-risk genes associated with non-syndromic ASD include *CNTNAP2, GRIN2B, CHD8, SCN2A, PTEN, and SHANK3*. Notably, SHANK3 mutations can also occur in ASD patients without Phelan-McDermid syndrome, indicating that a single gene mutation may lead to ASD alone. The overlapping region highlights shared molecular pathways, such as synapse formation, synaptic pruning, and ubiquitination, demonstrating that different genetic backgrounds converge on common synaptic mechanisms.

This review focuses on the ASDs driven by genetic factors, broadly categorized into syndromic and non-syndromic forms (Figure 1). The causative genes for neurological syndromes such as Fragile X syndrome, Rett syndrome, Angelman syndrome, and tuberous sclerosis have been identified as *Fragile X Mental Retardation 1* (*FMR1*)*, Methyl-CpG-binding Protein 2* (*MECP2*)*, Ubiquitin Protein Ligase E3A* (*UBE3A*)*, and Tuberous Sclerosis Complex* (*TSC*) *1 and TSC2*, respectively. These genes are implicated in synaptic development and function, transcriptional regulation, and ubiquitin-mediated processes, while TSC1/2 participates in signaling pathways mediated by synaptic adhesion molecules (Sztainberg and Zoghbi, 2016; Guang et al., 2018). These syndromes exhibit a high incidence of comorbid ASD symptoms. Furthermore, large-scale genomic analyses have identified numerous candidate genes with *de novo* mutations in non-syndromic ASD. Genes strongly associated with ASD onset include those involved in synaptic adhesion, signaling, and function, such as *Neuroligins* (*NLGN*), *Neurexins* (*NRXN*), *Contactin-associated protein-like 2* (*CNTNAP2*), *Cell adhesion molecule 1* (*CADM1*), and *SHANK*, as well as regulators of cell differentiation, including *DISC1* and *PTEN* (Guang et al., 2018). However, no single convergent pathway has been established among these genes, and the prevalence of mutations in each gene ranges from approximately 0.5% to 3% (Yasuda et al., 2023). This genetic heterogeneity underscores the complexity of ASD pathogenesis and the need for integrative approaches to identify shared molecular mechanisms related to ASD comorbidities.

Speech and language disorder (SLD) is diagnostically distinct from ASD; however, substantial phenotypic overlap exists in communication deficits in both syndromic and non-syndromic ASD (Konopka, 2012; Morgan et al., 2024). Recent evidence suggests that *Forkhead box P2* (*FOXP2*) is a key gene for speech and language development, and it may also contribute to ASD-related communication deficits through shared neurodevelopmental pathways (Haghighatfard et al., 2022). *FOXP2* encodes a transcription factor expressed in cortical, striatal, and cerebellar circuits, where it regulates genes involved in synaptic plasticity and neuronal differentiation. Mutations in *FOXP2* disrupt these processes, leading to impaired vocalization and language acquisition.

In this review, we highlight findings from animal models including mouse, zebra finches, zebrafish, and *Drosophila*, and focus on social interaction mediated by speech and language communication. We further explore how synaptic molecules and neural network in specific brain regions contribute to the relationship between *FOXP2*-related speech and language disorder (*FOXP2*-SLD) and communication impairments in ASD.

We also describe homozygous *Foxp2* R552H knock-in (*Foxp*2^R552H/R552H^) mice (Fujita et al., 2008; Fujita-Jimbo et al., 2025) which exhibit early lethality, and we discuss the possible relevance of FOXP2-related gastrointestinal abnormalities reported in these mutants, which may provide insights into ASD comorbidities. Finally, we propose some research on the brain neural circuits of animal models expressing *Foxp2*-promoter–mediated fluorescent probes and synaptic protein-promoter-mediated reporters as the future directions for research integrating genetic, neurodevelopmental, and behavioral perspectives to better understand *FOXP2*-mediated pathways in ASD.

## 2 Excitatory and inhibitory synapses in ASD

**Figure 2:**
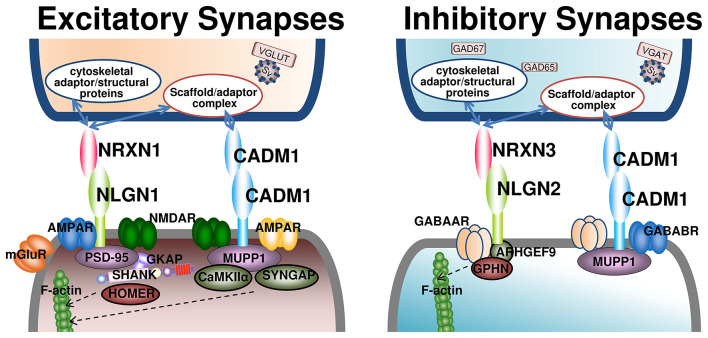
Key adhesion molecules at excitatory and inhibitory synapses and the importance of their balance. This figure summarizes major adhesion molecules and scaffold/adaptor proteins at excitatory and inhibitory synapses. At excitatory synapses **(left)**, presynaptic Neurexin1 binds postsynaptic Neuroligin1, with PSD-95 as a central scaffold; Cadm1 contributes to spine formation and synapse stabilization. At inhibitory synapses **(right)**, postsynaptic Neuroligin2 organizes an inhibitory scaffold centered on Gephyrin. Balanced excitatory and inhibitory synaptic organization is essential for normal circuit function and plasticity, and its disruption is implicated in neurological disorders. The presynaptic scaffold/adaptor complex includes CASK, Mint, and other synaptic organizing molecules. In contrast, cytoskeletal adaptor/structural proteins at presynaptic sites include Ankyrin-G, Spectrin, F-actin, and actin-associated protains. AMPAR, AMPA-type glutamate receptor; ARHGEF9, collybistin; Cadm1, cell adhesion molecule 1; CaMKIIα, Ca^2+^/calmodulin-dependent protein kinase II alpha subunit; CASK, calcium/calmodulin-dependent serine protein kinase; GABAAR, GABAA receptor; GABABR, GABAB receptor; GAD65/67, glutamate decarboxylase 65/67; GKAP, guanylate kinase–associated protein; GPHN, gephyrin; Mint, Munc18-interacting protein; mGluR, metabotropic glutamate receptor; MUPP1, multi-PDZ domain protein 1; NLGN, neuroligin; NMDAR, NMDA-type glutamate receptor; NRXN, neurexin; SV, synaptic vesicle; SynGAP, synaptic Ras GTPase-activating protein 1; VGAT, vesicular GABA transporter; VGLUT, vesicular glutamate transporter.

Synapses involved in information transmission are broadly classified into excitatory and inhibitory synapses (Figure 2 and Table 1). The balance between excitatory and inhibitory activity (E/I balance) is regulated by excitatory glutamatergic neurons and inhibitory GABAergic interneurons. This balance is critical during neural development, mediating the migration and positioning of pyramidal cells and interneurons to establish cortical organization (Manent and Represa, 2007). It is also essential for normal brain function, underpinning cognition and behavior (Canitano and Pallagrosi, 2017; Sohal and Rubenstein, 2019).

**Table 1:** Synaptic molecules implicated in autism spectrum disorder.

**Synapse type**	**Molecule**	**Classification (role)**	**ASD genetics (SFARI-based)**	**Function/E/I relevance**	**Key references**
Excitatory/inhibitory	NLGN1 (Neuroligin-1), NLGN2 (Neuroligin-2), NLGN3/4 (Neuroligin-3/4X)	Postsynaptic adhesion molecule	+++ (NLGN1: SFARI score 2; NLGN2: SFARI score 1; NLGN3: strong ASD-linked variants; NLGN4X: SFARI score 1)	Adhesion regulating both excitatory and inhibitory synapses; affects E/I balance	Jamain et al., 2003; Chih et al., 2005; Chubykin et al., 2007
Excitatory/inhibitory	NRXN11/2/3 (Neurexin-1/2/3)	Presynaptic adhesion molecule	+++ (NRXN1/2/3, SFARI Score 1; NRXN1 has repeated *de novo* CNVs/LoF)	Presynaptic adhesion; organizes E/I circuitry	Powell and Boucard, 2010
Excitatory/inhibitory	CADM1	Synaptic adhesion molecule	++ (SFARI score 2)	Drives synapse formation	Biederer et al., 2002; Fujita et al., 2010, 2012
Excitatory/inhibitory	CNTNAP2	Adhesion molecule (Neurexin family)	++ (SFARI score 2S)	Circuit abnormalities; reduced E/I synaptic input	St. George-Hyslop et al., 2022; Lazaro et al., 2019
Transcriptional/circuit-wide	FOXP1	Transcription factor	+++ (SFARI score 1S)	Regulates synaptic gene programs communication-related circuitry	Anderson et al., 2020; Ortiz et al., 2025
Transcriptional/circuit-wide	FOXP2	Transcription factor	+++ (SFARI score 1)	Controls CNTNAP2/VLDLR synaptic pathways	Bowers and Konopka, 2012
Transcriptional/circuit-wide	FOXP4	Transcription factor	— (No SFARI ASD scoring)	Co-regulates synaptic genes with FOXP1/2 [cell.com]	Bowers and Konopka, 2012
Inhibitory	MUPP1	PDZ scaffold	— (No SFARI ASD scoring)	Links CADM1 to inhibitory receptor complexes	Fujita et al., 2012
Excitatory	SHANK3	PSD scaffold	+++ (SFARI score 1S)	Core PSD scaffold for excitatory synapses	Powell and Boucard, 2010
Excitatory	SHANK2	PSD scaffold	+++ (SFARI score 1)	PSD structural and signaling scaffold	Powell and Boucard, 2010
Excitatory	SHANK1	PSD scaffold	++ (SFARI score 2)	Stabilizes excitatory PSD	Powell and Boucard, 2010
Excitatory	PSD-95 (DLG4)	PSD scaffold (MAGUK)	+++ (SFARI score 1)	Central organizer for excitatory PSD; receptor clustering, E/I effects	Chih et al., 2005
Excitatory	HOMER1/2/3	PSD scaffold	+ (Homer1: SFARI score 2, Homer2/3: suggestive; none is high confidence)	Scaffold for mGluR5–Shank complex	Powell and Boucard, 2010
Inhibitory	ARHGEF9 (Collybistin)	Gephyrin recruiter (GEF)	+++ (SFARI lists ARHGEF9 as score 1S)	Inhibitory postsynapse assembly; GPHN/GABA_A clustering	Papadopoulos and Soykan, 2011
Inhibitory	GPHN (Gephyrin)	Inhibitory postsynaptic scaffold	+ (SFARI score 2)	GABA/Gly receptor clustering	Papadopoulos and Soykan, 2011; Lionel et al., 2013

Evidence codes indicate the strength of ASD-related genetic support: “—” denotes no established ASD evidence; “+” indicates suggestive or associated reports; “++” reflects established ASD-linked variants or CNVs; and “+++” corresponds to high-confidence ASD genes supported by recurrent or robust findings (e.g., *de novo* loss-of-function or replicated CNVs). These codes follow the SFARI Gene scoring framework (https://gene.sfari.org/about-gene-scoring/). CNV, Copy Number Variations; LoF, Loss-of-Function.

Disruption of E/I balance has been implicated in ASD, although the specific brain regions and neural networks affected remain insufficiently characterized (Canitano and Palumbi, 2021; Si and Zhang, 2025). Evidence from animal models supports this hypothesis (Fang et al., 2014; Gonçalves et al., 2017; Lee et al., 2017; Perera-Murcia et al., 2026). Studies using animal models have demonstrated that behavioral and circuit abnormalities arising from diverse ASD-associated risk genes often converge on common neurophysiological phenotypes, such as weakened inhibitory circuits and excessive strengthening of excitatory synapses (Lee et al., 2017). *In vivo* MRS analyses further indicate that ASD mouse models exhibit region-specific patterns of E/I imbalance across the brain (Gonçalves et al., 2017). Additionally, excessive formation of upper-layer cortical neurons has been shown to increase circuit excitability and induce ASD-like behaviors (Fang et al., 2014). In the *Shank*3^+/−^ model, disruptions in mGluR- and NMDA-dependent synaptic plasticity have been linked to cognitive impairments (Perera-Murcia et al., 2026). These findings suggest that the molecular underpinnings of E/I dysregulation in ASD are gradually being elucidated, and that both syndromic and non-syndromic ASD may share convergent pathways involving synaptic development and E/I balance.

### 2.1 Convergent synaptic adhesion pathways

Synaptic adhesion molecules play a critical role in establishing and maintaining synaptic connectivity, and their dysfunction has been strongly implicated in ASD pathophysiology.

#### 2.1.1 Neurexin-Neuroligin, *Cadm1*, and ASD

Neuroligin family (NLGN1, NLGN3, NLGN4): Neuroligins are postsynaptic adhesion proteins that interact with presynaptic Neurexins to stabilize synaptic contacts. Neuroligin-3 and Neuroligin-4 knockout (KO) mice, Neuroligin-3 R451C mutant mice, KO mice for Neurexin-1, -2, and -3 (adhesion partners of Neuroligin), as well as Shank3 KO mice, display phenotypes such as impaired social interaction and repetitive behaviors (Tabuchi et al., 2007; Durand et al., 2007; Zhang et al., 2009). *NRXN1* deletions and missense mutations are among the most frequently reported genetic alterations in ASD, leading to disrupted synaptic connectivity and altered neurotransmission (Südhof, 2008).

Similarly, mutations in other synaptic adhesion proteins such as *CNTNAP2* and *CADM1* are associated with ASD symptoms and related neurodevelopmental phenotypes (St. George-Hyslop et al., 2022; Zhiling et al., 2008). *CADM1* is an immunoglobulin superfamily member acting as a synaptic adhesion molecule similar to Neuroligin and CNTNAP2 (Urase et al., 2001; Biederer et al., 2002; Fujita et al., 2010). Two missense mutations (H246N, Y251S) identified in Japanese ASD patients cause membrane trafficking defects and endoplasmic reticulum stress, leading to dendritic shortening and impaired synaptogenesis (Zhiling et al., 2008; Fujita et al., 2010). Furthermore, CADM1 undergoes ectodomain shedding, with ADAM10 mediating its endogenous cleavage and ASD-associated CADM1 missense variants (H246N, Y251S) showing increased susceptibility to this proteolysis; moreover, neuronal RA175/SynCAM1 (CADM1) isoforms can be processed by Tumor Necrosis Factor-α Converting Enzyme (TACE)/A disintegrin and metalloprotease domain (ADAM)17-like proteases, suggesting that isoform- and mutation-dependent proteolysis may reduce synaptic-surface CADM1 (Nagara et al., 2012; Zhiling et al., 2008; Tanabe et al., 2008). At the circuit/behavioral level, *Cadm1*-knockout mice show impaired social and emotional behaviors (Takayanagi et al., 2010), and CADM1 forms cerebellar synaptic complexes with Multi-PDZ Domain Protein 1 (MUPP1), a multivalent PDZ scaffold protein (Fujita et al., 2012). Notably, CADM1 has also been implicated in ADHD-related traits in both mouse and human studies (Sandau et al., 2012; Jin et al., 2019). Importantly, *Cadm1*-deficient pups exhibit communication-related phenotypes, including impaired ultrasonic vocalizations and smaller cerebella (Fujita et al., 2012). These findings link CADM1-dependent synaptic dysfunction to communication-related phenotypes, including impaired ultrasonic vocalizations.

Furthermore, ASD-associated mutations in *Neuroligin-3* have been shown to impair developmental synapse elimination in the cerebellum, specifically the pruning of climbing fiber inputs (Lai et al., 2021). This supports the possibility that disrupted maturation of cerebellar circuits may contribute to ASD-relevant communication phenotypes. Consistent with this view, multiple ASD-related genes have been implicated in the molecular and cellular mechanisms governing cerebellar synapse elimination, suggesting convergence on shared developmental processes at the circuit level despite marked genetic heterogeneity (Watanabe and Kano, 2024).

#### 2.1.2 Mutated CNTNAP2 Functions related to ASD

*CNTNAP2* mutations disrupt cortical excitation/inhibition (E/I) balance, contributing to aberrant neural development and ASD-like behaviors (Peñagarikano et al., 2011; Selimbeyoglu et al., 2017). In *CNTNAP2* knockout mice, abnormalities in neuronal migration during cortical development and spontaneous seizures have been reported (Peñagarikano et al., 2011), suggesting that the loss of CNTNAP2 function at the circuit formation level leads to extensive network abnormalities. It has been reported that correcting the excitatory/inhibitory (E/I) balance in the prefrontal cortex optogenetically significantly improves sociability in *CNTNAP2* knockout mice, strongly indicating that the skewing of the E/I ratio in local circuits of the prefrontal cortex is a major factor in social behavior abnormalities (Selimbeyoglu et al., 2017).

Knockdown of *CNTNAP2* in layer 2/3 pyramidal neurons of the mouse prefrontal cortex reduces excitatory and inhibitory synaptic transmission, impairs social interaction, and induces mild pup vocalization abnormalities, consistent with synaptic mechanisms contributing to ASD-like phenotypes (Sacai et al., 2020). Beyond the cortex, recent studies further show that CNTNAP2 loss drives striatal hyperexcitability and repetitive, inflexible behaviors, reinforcing its role in ASD pathophysiology (Cording et al., 2025), and is also associated with molecular pathway disruptions in the prefrontal cortex (Jang et al., 2023).

These studies demonstrate that *CNTNAP2* mutations converge on ASD-like phenotypes through multiple hierarchical mechanisms including altered cortical development, disrupted E/I balance in prefrontal circuits, and striatal hyperexcitability. Evidence that circuit-level E/I manipulation can rescue social deficits (Selimbeyoglu et al., 2017), together with synaptic and behavioral effects of CNTNAP2 disruption in prefrontal pyramidal neurons (Sacai et al., 2020), supports a central role for CNTNAP2 in regulating neural circuit function relevant to ASD.

## 3 Mutated Forkhead-box Protein P2 (FOXP2) in SLD and ASD

Although ASD and SLD are diagnostically distinct, they share considerable phenotypic overlap in communication deficits; individuals with ASD often exhibit difficulties in using communicative language and producing speech (Lai et al., 2001; Vargha-Khadem et al., 2005). A missense mutation (R553H) in the *FOXP2* gene, located on 7q31 (SPCH1), a chromosomal region that has also been implicated by linkage studies in ASD (AUTS1), was identified in affected members of the KE family, where severe SLD is transmitted as an autosomal-dominant monogenic trait (Lai et al., 2001). The R553H mutation severely disrupts DNA-binding and transcriptional activity of *FOXP2*, resulting in orofacial motor impairment and monogenic speech-language disorders affecting articulation and motor skills (Lai et al., 2001; Vernes et al., 2008; Fisher and Scharff, 2009).

FOXP2 forms heteromeric complexes with Forkhead box P1 (FOXP1) and Forkhead box P4 (FOXP4; Li et al., 2004) and interacts with transcriptional regulators such as TBR1 and β-catenin and regulates the expression of numerous genes implicated in ASD, including *SRPX2, VLDLR*, and *CNTNAP2*, thereby influencing synaptic plasticity and neurodevelopment relevant to speech and language (Roll et al., 2010; Mendoza and Scharff, 2017; den Hoed et al., 2021). Furthermore, *FOXP2* and *FOXP1* mutations have been also implicated in a subset of ASD cases (Bowers and Konopka, 2012; Hamdan et al., 2010; Haghighatfard et al., 2022). The transcription factor Foxp proteins function as upstream regulators of synapse-related pathways and contribute to the modulation of gene networks that govern synaptogenesis and neural circuit formation.

*CNTNAP2*, a direct transcriptional target of FOXP2, is highly expressed in cortical circuits essential for language development and processing (Rodenas-Cuadrado et al., 2014; Vernes et al., 2008; Adam et al., 2017). *FOXP2*-mediated transcriptional networks, including regulation of *CNTNAP2*, influence synaptic architecture and connectivity (Mendoza and Scharff, 2017). Synaptic abnormalities and disrupted interregional connectivity in specific brain regions may contribute to ASD pathogenesis; ASD may arise from dysfunction in specific brain circuits rather than global brain impairment (den Hoed et al., 2021; Oswald et al., 2017; Mukamel et al., 2011; Valle-Bautista et al., 2024). *FOXP2* encodes a nuclear transcription factor implicated in cognitive functions across humans, non-human mammals, and song-learning birds (Enard et al., 2002).

### 3.1 FOXP2 networks and brain region-specific mechanisms

*FOXP2* dysfunction may impact molecular networks underlying vocal learning and converge with ASD-related pathways (den Hoed et al., 2021). In mice, ultrasonic vocalizations (USVs) serve as an analog of human speech, and their patterns are analyzed in social contexts such as male–female interactions and maternal–offspring communication. *Foxp*2^+/R552H^ mice, modeling the human R553H mutation, survive and exhibit altered USV patterns together with (Fujita et al., 2008), whereas *Foxp2*-deficient mice and *Foxp*2^R552H/R552H^ mice exhibit severe USV deficits with abnormal Purkinje cell development and early postnatal lethality within 3–4 weeks after birth (Shu et al., 2005; Fujita et al., 2008).

Reciprocal connectivity between the thalamus and cortex occurs via thalamocortical fibers and corticothalamic fibers (Bandiera and Molnár, 2022; Ding, 2024); core relay neurons from a given thalamic nucleus project topographically to a sharply defined architectonic field in layer IV of the cortex, corresponding to sensory modalities such as vision, audition, and somatosensation (e.g., the barrel field; Ding, 2024; Bertero et al., 2022). In contrast, two major corticofugal projection neurons arise in the cortex: corticothalamic neurons in layer VI and subcerebral projection neurons in layer V (Bandiera and Molnár, 2022; Ding, 2024). FOXP2 is prominently expressed in cortical layers VI and V (Qi et al., 2024; Ebisu et al., 2017).

Recently, Druart et al. (2020) showed that *Foxp*2^+/R552H^ affects the complexity of apical dendrites of Layer VI neurons in the auditory cortex, reduces excitatory synaptic inputs, and decreases intrinsic excitability through increased GABA_B_/GIRK signaling. *FOXP2*, as a transcriptional regulator of neurodevelopmental genes, regulates the expression of *VLDLR*, a Reelin receptor implicated in ASD (Adam et al., 2016; Mendoza and Scharff, 2017), which controls the migration of cortical neurons and their neuronal invasion into the marginal zone of the cortical layer (Hirota and Nakajima, 2020). FOXP2 may intersect with these pathways, providing a unique perspective on communication deficits in ASD. While FOXP2 also directly regulates *CNTNAP2* (Vernes et al., 2008), other adhesion molecules converge on similar neurodevelopmental pathways, influencing synapse formation, plasticity, and E/I balance (Lee et al., 2017; Gonçalves et al., 2017; Perera-Murcia et al., 2026). ASD arises from multi-layered disruptions in synaptic architecture and circuit formation (Sohal and Rubenstein, 2019; Fang et al., 2014; Si and Zhang, 2025). These convergent pathways suggest that *FOXP2*-related mechanisms intersect with synaptic adhesion networks, forming a molecular basis for communication deficits in ASD and SLD.

### 3.2 Development of thalamic nuclei and expression of *Foxp2* and *Cadm1*

The thalamus develops in the posterior diencephalon, between the mesencephalon and telencephalon. It serves as the primary relay center for sensory signals to the cortex. The adult thalamus comprises multiple anatomically and functionally distinct nuclei, categorized by their position (anterior, medial, lateral, ventral, posterior, and intralaminar groups), cytoarchitecture, input type (sensory, motor, or limbic), and hierarchical connectivity patterns with the cerebral cortex and subcortical regions (Jones, 2007; Segobin et al., 2024).

FOXP2 expression is low in the anterior region of the thalamic primordium. In *Foxp*2^R552H/R552H^ mice, posterior thalamic nuclei are reduced in size, intermediate nuclei are expanded, and cortical barrel fields are disrupted, corresponding to thalamic shrinkage (Ebisu et al., 2017). The secreted morphogen Sonic Hedgehog (Shh) plays a critical role in thalamic development through spatiotemporal and threshold-dependent mechanisms. Shh regulates the differentiation of FOXP2-positive and CADM1-positive progenitor cells during thalamic development (Govek et al., 2022). Because thalamic organization is critical for sensory processing, these developmental abnormalities may cause sensory deficits, including auditory impairments associated with autism spectrum disorder (Kurt et al., 2012; Zheng and Chen, 2025).

**Figure 3:**
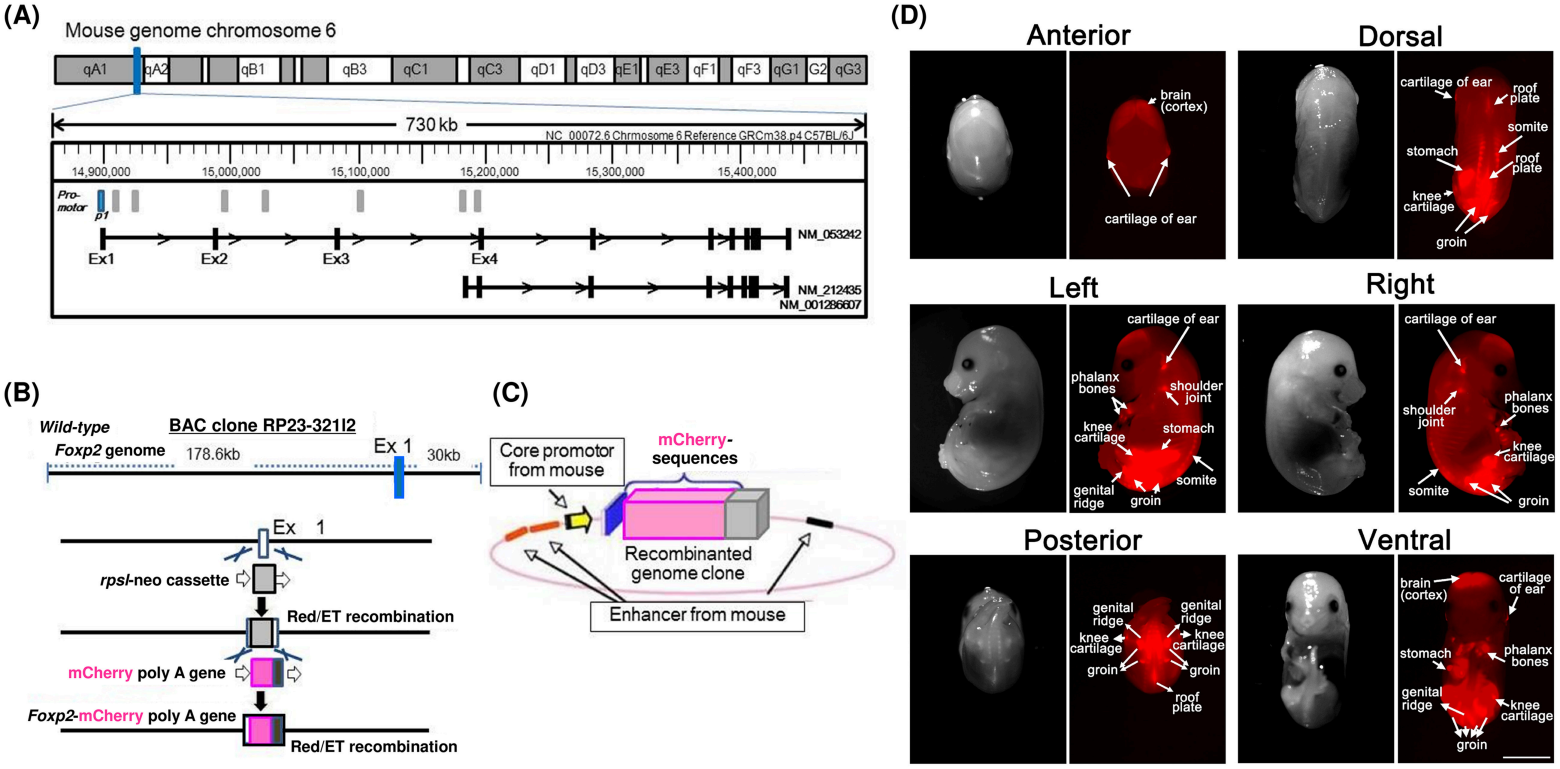
Detection of mCherry in Foxp2 promoter-mediated mCherry-Tg (*Foxp*^2+/+^*/*mCherry-Tg) mice. **(A)** Production of mCherry-Tg. Mouse Foxp2 is located on the long arm of chromosome 6. **(B)** The bacterial artificial chromosome (BAC) clone (No. RP23-321I2) spans 178.76 kb upstream and 30 kb downstream of *Foxp2* exon 1. Red/ET recombination was performed, and the mCherry-poly(A) gene was inserted into the clone. **(C)** A recombinantly engineered BAC clone containing the Foxp2 gene with its native promoter. **(D)** Images from six positions of the whole body at embryonic day 13.25. Red indicates mCherry. Positive signals are visible in the brain and cartilage of the ear (anterior view); in the brain, cartilage of the ear, phalanx bones, shoulder joint, stomach, knee cartilage, genital ridge, groin, and somites (left view); in the brain, groin, knee cartilage, genital ridge, and roof plate (posterior view); in the cartilage of the ear, stomach, knee cartilage, groin, roof plate, and somites (dorsal view); in the cartilage of the ear, phalanx bones, shoulder joint, knee cartilage, groin, and somites (right view); and in the brain, cartilage of the ear, phalanx bones, knee cartilage, groin, genital ridge, and stomach (ventral view). Strong mCherry positivity is observed in the stomach and groin. Scale bar: 2 mm. Figure reproduced from Fujita-Jimbo et al. (2025) under CC BY-NC-ND 4.0.

The more impaired USVs observed in *Foxp*2^R552H/R552H^ mutants may partly reflect disrupted thalamocortical circuit formation associated with abnormal morphology of the thalamus and cortex during development (Ebisu et al., 2017; Druart et al., 2020). However, Foxp2-positive neural network cannot be directly visualized because FOXP2 is a nuclear protein with transcriptional activity. Recently, we developed Foxp2-promoter-mediated mCherry-Tg (mCherry-Tg) mice to visualize FOXP2-positive tissues and FOXP2-positive brain regions and putative projection patterns during development (Figure 3; Fujita-Jimbo et al., 2025). Future studies should integrate genetic, neurodevelopmental, and behavioral perspectives to clarify these interactions and identify therapeutic targets. *Foxp*2^+/+^/mCherry-Tg and *Foxp*2^R552H/R552H^/mCherry-Tg mice will be useful for mapping the expression of ASD risk synaptic adhesion molecules, such as CNTNAP2 and CADM1, within the FOXP2-positive neural circuits in the specific brain regions, including the auditory system.

## 4 Abnormal brain and gastrointestinal (GI) development of homozygous *Foxp2* R552H-knock-in mice

As shown in Figure 3, *Foxp2* is expressed in various tissues during development, including the brain and GI system, and is associated with morphological abnormalities in the cerebellum, thalamus, and GI system (Fujita et al., 2008; Ebisu et al., 2017; Fujita-Jimbo et al., 2025).

Unlike *Foxp*2^+/R552H^ mice, *Foxp*2^R552H/R552H^ mice do not survive beyond 3–4 weeks, and morphological abnormalities are detectable during embryonic development and the early postnatal period (Fujita et al., 2008). *Foxp*2^R552H/R552H^ mice also exhibit abnormal GI tract development, including atrophy of the stomach, abnormal intestine rotation, and gut necrosis (Fujita-Jimbo et al., 2025).

In humans, the phenotype associated with heterozygous FOXP2 R553H mutation primarily involves orofacial motor impairment affecting speech articulation and motor skills (Vargha-Khadem et al., 2005). However, homozygous R553H mutation has not been reported, indicating a translational gap between mouse and human pathology. Furthermore, only limited studies have systematically evaluated gastrointestinal symptoms or motility in individuals with *FOXP2*-related speech and language disorders. As current clinical reviews do not report gastrointestinal involvement, this remains an open question that should be addressed through future phenotype expansion efforts, including the standardized assessment of gastrointestinal features. Although no overt gastrointestinal symptoms have been described in heterozygous *FOXP2* mutation carriers, subtle or subclinical gastrointestinal motility alterations may have been under-recognized due to the absence of systematic assessment. Considering the widespread FOXP2 expression in developing gut tissues, such mild phenotypes warrant further investigation.

To visualize FOXP2 expression, we generated transgenic (*Foxp*2^+/R552H^/mCherry-Tg and *Foxp*2^R552H/R552H^/mCherry-Tg) mice (Figure 3). mCherry fluorescence was detected in the brain, neural crest, GI tract, stomach, and somites of *Foxp*2^+/+^/mCherry-Tg embryos at E12.5 and in pups at P12, supporting the spatiotemporal distribution of FOXP2 promoter activity and providing an entry point to investigate its role in brain–gut axis development (Fujita-Jimbo et al., 2025). This section examines GI abnormalities associated with FOXP2 mutations and their relevance to the brain–gut axis as characterized in the model mice.

### 4.1 Gastrointestinal (GI) disorders and Hirschsprung-like phenotypes

GI abnormalities such as obstructive ileus have also been reported in other neurological disorders, including Rett syndrome (Caputi et al., 2024; Berger et al., 2024) and major depressive disorder (Kim et al., 2023), suggesting that GI comorbidities are common across neurodevelopmental and neuropsychiatric conditions. Individuals with ASD frequently exhibit GI abnormalities, including motility dysfunction and increased intestinal permeability, and a higher prevalence of inflammatory bowel disease and other GI disorders (Hsiao et al., 2013; Kohane et al., 2012; Holingue et al., 2018; McElhanon et al., 2014). The brain–gut–microbiome axis has been proposed as a potential pathogenic mechanism underlying neurodevelopmental disorders, including ASD (Hsiao et al., 2013; Wang et al., 2023).

GI motility and digestion are coordinated by epithelial and smooth muscle tissues under the control of the enteric nervous system (ENS), comprising myenteric and submucosal plexuses derived from migrating enteric neural crest cells (ENCCs). ENS development relies on ENCC migration, proliferation, and differentiation from anterior to posterior gut regions (Lake and Heuckeroth, 2013; Burns and Goldstein, 2024). As the ENS integrates motility and interfaces with the epithelial barrier, immunity, and secretion, developmental disruption can alter intestinal permeability and inflammatory tone, impacting gut–brain interactions (Lake and Heuckeroth, 2013; Wang et al., 2023). Hirschsprung disease is caused by failed ENCC colonization, resulting in distal colonic aganglionosis and functional obstruction (Burns and Goldstein, 2024). Although ASD-associated GI and microbiome phenotypes vary across individuals, animal studies show that increased permeability and altered metabolites can accompany or contribute to behavioral changes; however, causal relationships remain difficult to establish (Hsiao et al., 2013; Wang et al., 2023). Given that Hirschsprung-like phenotypes involve distal colonic aganglionosis, future work should assess enteric plexus formation across developmental stages and characterize niche interactions within the distal colon (Burns and Goldstein, 2024; Zhou et al., 2024).

*Foxp*2^R552H/R552H^ mice show severe GI developmental defects, including gastric atrophy, intestinal malrotation, gut necrosis, accompanied by reduced expression of Wnt/β-catenin antagonists such as *BarH-like homeobox 1* (*Barx1*) and *Secreted frizzled-related protein 1* (*Sfrp1*; Fujita-Jimbo et al., 2025). They also show impaired epithelial integrity (decreased E-cadherin and Zonula occludens-1) and elevated serum zonulin, consistent with increased intestinal permeability (“leaky gut”), as well as reduced α-smooth muscle actin (α-SMA), suggesting smooth-muscle atrophy (Fujita-Jimbo et al., 2025). Altered expression of ENS-related genes (*Ret, Phox2b*) further suggests disruption of epithelial–smooth–muscle niches and neural-crest–derived ENS development. Consistent with this, *Foxp2* ^R552H/R552H^/mCherry-Tg mice exhibit reduced ENS innervation in the stomach. Whether these changes extend to the distal colon and produce a Hirschsprung-like phenotype remains to be determined through systematic evaluation of distal colonic ganglia across development (Fujita-Jimbo et al., 2025; Burns and Goldstein, 2024).

*Foxp*2^+/+^ and *Foxp*2^R552H/R552H^/mCherry-Tg mice provide a useful framework and will also be valuable for future studies to distinguish direct from indirect effects of *Foxp2* on GI development, including potential FOXP2 binding to regulatory regions of key gut-development genes such as *Barx1*, and to investigate FOXP2-expressing tissues in relation to the brain–gut axis in ASD.

Overall, *Foxp*2^R552H/R552H^ mice offer a tractable platform for linking gut morphogenesis, barrier and ENS abnormalities, and gut–brain interactions. While microbiome and ENS consequences remain incompletely characterized, current findings suggest disruption of Wnt/β-catenin–related pathways during gut development (Fujita-Jimbo et al., 2025). Beyond the authors' mouse line, future work should integrate evidence across species (*zebrafish, songbirds*) and human-relevant systems (organoids and patient-derived data) to resolve conserved vs. species-specific FOXP2–gut mechanisms.

## 5 Other model animals for communication neuronal network related to ASD

In addition to mice, which are widely used to investigate mammalian social behavior and cortical circuits and provide a powerful model for studying *FOXP2*-dependent vocal learning pathways, *Drosophila* offers a uniquely tractable system for dissecting ASD-associated genes across molecular, circuit, and behavioral levels (Bellosta and Soldano, 2019; Tian et al., 2017; Castells-Nobau et al., 2019). Zebrafish complement these models by enabling whole-brain analyses of development, neural activity, and social behavior at high spatiotemporal resolution (Rea and Van Raay, 2020; Pal et al., 2025). Together, these systems allow for an integrated investigation of ASD phenotypes from molecular mechanisms to higher-order behaviors.

### 5.1 FoxP2 in the song neuronal network in zebra finches

This section outlines the role of FOXP2 in the vocal learning circuits of zebra finches, emphasizing evolutionary conservation and its relevance to communication disorders. The zebra finches are valuable animal models for studying song-related neuronal networks. Area X, a striatal nucleus in the zebra finch brain, is part of the specialized song system (anterior forebrain pathway) involved in song acquisition, vocal learning, and the refinement of stereotyped courtship songs through auditory feedback (Thompson et al., 2007). *FOXP2* is expressed in newly generated spiny neurons within adult Area X, where it regulates spine dynamics and influences song plasticity (Schulz et al., 2010). FOXP2 contributes to structural plasticity of dendritic spines, as demonstrated by synaptic plasticity studies in *FOXP2* mouse models (Schulz et al., 2010). Reduction of *FOXP2* impairs vocal learning and alters dopaminergic modulation of corticostriatal signaling, which is critical for song variability and involves developmental regulation of dopamine D1 receptor expression (Murugan et al., 2013; Co et al., 2020). Comparative studies in songbirds highlight the evolutionary conservation of FOXP2 function in vocal learning (Pfenning et al., 2014), offering insights into communication deficits observed in ASD.

FOXP2 interacts with dopamine signaling in corticostriatal circuits to regulate song variability and learning. Reduced FOXP2 levels disrupt dopaminergic modulation of corticostriatal signaling (Murugan et al., 2013), while FoxP family proteins regulate target genes such as *VLDLR* and *CNTNAP2* in the zebra finch song system (Mendoza and Scharff, 2017); FOXP2 binds to the promoter of *VLDLR*, one of the two receptors for Reelin, and activates its transcription, whereas FoxP2 knockdown in Area X of zebra finches downregulates *VLDLR* expression. Additionally, cortical FOXP2 supports behavioral flexibility and dopamine D1 receptor development (Co et al., 2020).

### 5.2 Zebrafish models of ASD

Zebrafish models offer unique advantages for studying ASD-related genes, including optical transparency for live imaging, rapid development, and suitability for high-throughput drug screening (Pal et al., 2025; Rea and Van Raay, 2020; Lüffe et al., 2021). Various gene mutations have been reported as zebrafish models of ASD (Pal et al., 2025). *shank3* mutations disrupt synaptic structure and function, leading to neural and behavioral deficits in zebrafish ASD models (Kozol et al., 2021; Liu et al., 2018). A mutation of the zebrafish gene associated with Rett Syndrome, *mecp2*, results in mutant larvae that spend more time toward the center of the tank than their siblings (Santistevan et al., 2024). In *cntnap2*-mutant fish, abnormal interactions between neurons and glia and altered synaptic development have been reported (Hoffman et al., 2016). Zebrafish with a mutation in the neurexin2aa gene, a homolog of a synaptic protein-encoding gene associated with ASD (*NRXN2*), exhibit increased anxiety by remaining at the bottom of a novel tank longer than heterozygous siblings (Koh et al., 2021). Environmental context has been shown to modulate sociability in *ube3a* zebrafish mutants (Angelman syndrome model) through alterations in sensory pathways, enabled by an approach integrating visual input with behavioral analysis to dissect social communication (Dougnon and Matsui, 2026). Although these genetic models vary in behavioral entry points, they converge on shared neurobiological mechanisms, including synaptogenesis, synaptic plasticity, and regulation of E/I balance. ASD-associated genes and molecular pathways represented in these zebrafish models are summarized in Table 2.

**Table 2:** ASD-related genes and behavioral phenotypes in zebrafish.

**Gene/model**	**Method**	**Associated disorder**	**Developmental stage**	**Behavioral phenotype**	**Primary reference (full)**
foxP2	Zinc-finger nuclease (ZFN) mutant/enhancer regulation study CRISPR/Cas9	Neurodevelopmental Disorder ASD ADHD	Embryo Embryo Larva	No behavioral phenotype; ZFN mutants show no axon guidance or CNS defects./No behavioral phenotype; study focused on regulatory/enhancer Locomotor activity via GABAergic signaling	Xing et al., 2012 (PLoS ONE 7: e43968); Bonkowsky et al., 2008; Lüffe et al., 2021
shank3a/shank3b	CRISPR	ASD; Phelan–McDermid	Larva, Adult	Reduced social interaction; repetitive swimming; dampened dark-flash response	Liu et al., 2018; Kozol et al., 2021
shank3 (Morpholino)	Morpholino (Knockdown)	ASD; Phelan–McDermid	Larva	Developmental delay; seizure-like behaviors; reduced GABAergic neurons	Kozol et al., 2015
syngap1 (Morpholino)	Morpholino (Knockdown)	ASD; ID	Larva	Developmental delay; seizure-like behaviors; reduced GABAergic markers	Kozol et al., 2015
cntnap2a/cntnap2b	CRISPR/Mutant	ASD; Epilepsy	Larva, juvenile	Hyperactivity; reduced social preference; altered sleep patterns	Hoffman et al., 2016
neurexin2aa	CRISPR	ASD	Embryo, larva, adult	Adult zygotic mutants exhibit increased anxiety	Koh et al., 2021
arid1b	CRISPR	ASD; Coffin–Siris	Larva	Sleep and social behavior alterations in arid1b mutants	Doldur-Balli et al., 2023
chd8	CRISPR	ASD	Larva	Macrocephaly tendency; social deficits; anxiety-like behavior	Wang et al., 2025
fmr1	ENU mutant/Knockout	Fragile X; ASD comorbidity	Larva, adult	Social and learning deficits; fine motor anomalies	Vaz et al., 2019
fmr1 (anxiety study)	ENU mutant/Knockout	Fragile X; ASD comorbidity	Larva	Increased anxiety-like behavior; molecular stress pathway changes	Dougnon and Matsui, 2025
ube3a	CRISPR or Mutant	ASD; Angelman syndrome	Larva	Increased anxiety-like behavior; altered sensory pathway gene expression	Dougnon and Matsui, 2025, 2026
ube3a+fmr1 (double)	CRISPR or Mutant	ASD; Angelman + Fragile X	Larva	Enhanced anxiety-like phenotype; synergistic stress pathway changes	Dougnon and Matsui, 2025
scn1lab	Mutant	Dravet syndrome; ASD comorbidity	Larva	Spontaneous electrographic seizures; convulsive swim; hyperactivity	Baraban et al., 2013
grin2b	CRISPR	ASD; ID	Larva, juvenile	Reduced social preference with preserved locomotion/learning	Zoodsma et al., 2022
chd2	CRISPR	ASD; epileptic encephalopathy	Larva	Photosensitivity; seizure susceptibility; activity changes	Li et al., 2024
mecp2 (null)	Null mutant	Rett syndrome; ASD features	Larva	Altered spontaneous and sensory-evoked motor behaviors; thigmotaxis defects	Pietri et al., 2013
mecp2 (behavior/transcriptome)	Mutant	Rett syndrome	Larva	Visual stimulus response alterations; thigmotaxis changes; conserved transcriptional effects	Santistevan et al., 2024
pten (ptena/ptenb)	Target-selected inactivation	ASD (macrocephaly association)	Larva, adult	Enhanced proliferation; survival; ocular tumors (ptenb^−/−^ adults)	Faucherre et al., 2008
Valproic acid (VPA)	Pharmacological	ASD-like induced phenotype	Larva, juvenile, adult	Reduced social preference; increased anxiety-like behavior; sleep/visual alterations	Camussi et al., 2024

*Foxp2* homologs in zebrafish provide a valuable framework for examining convergent mechanisms because they function as transcriptional regulators that integrate motor output, learning, and neurotransmission. CRISPR/Cas9-generated foxp2 mutants exhibit reduced GABAergic signaling and increased locomotion (Lüffe et al., 2021). In contrast, *foxp2* ZFN mutant zebrafish show normal axon pathfinding, indicating that not all FOXP2-linked neurodevelopmental readouts are consistently observed across zebrafish models (Xing et al., 2012). Comparative analyses will resolve these discrepancies.

Wnt/β-catenin signaling is positioned to influence ENS development because it governs early developmental gene programs, including those relevant to neural crest cell (NCC) specification and migration, as well as gastrointestinal formation (Sutton et al., 2021). In zebrafish, *lef1*, a Wnt-activated LEF/TCF family transcription factor, is necessary for embryonic *foxp2* expression and regulates *Lef1*-dependent *foxp2* enhancers, making *foxp2*-mutant and *foxp2*-promoter–based zebrafish lines well suited to test Wnt/β-catenin–dependent mechanisms underlying Hirschsprung-like, ENS-related outcomes because destruction of *Lef1/Tcf1* consensus sequence alters the *foxp2* expression in the brain (Bonkowsky et al., 2008). The *foxp2*-mutated and *foxp2*-promoter mediated models could also be used to test the Wnt/β-catenin hypothesis specifically in the context of *Foxp2*-mutated Hirschsprung-like pathology and ENS-related outcomes in future studies.

Zebrafish serve as valuable models for studying human diseases, including those related to the brain and nervous system (MacRae and Peterson, 2015). CRISPR-Cas9 technology for gene editing in zebrafish models can create models of human ASD mutants and mutants on candidate molecules related to synaptic function. Dystrophin is known as a candidate causative molecules for ASD (Rihel and Schier, 2012). The *dystrophin*-null mutant zebrafish, sapje, is an excellent model for Duchenne muscular dystrophy (DMD), suitable for analyzing the pathomechanisms of DMD and for therapeutic drug screening (MacRae and Peterson, 2015; Kawahara et al., 2011). Zebrafish produce large numbers of offspring, and their larvae can be cultured and treated with drugs in small wells, making them useful for high-throughput drug screening (Howe et al., 2013). Zebrafish models might have potential to discover new ASD treatments.

In the future, the development of various zebrafish models of ASD to cover a broad range of candidate molecules related to synaptic function and therapeutic drug screening using these models, might provide new ways to analyze the pathomechanism of ASD and identify treatments for ASD and disorders involving these candidate molecules.

### 5.3 *Drosophila* models of ASD

*Drosophila* melanogaster is a well-established model organism for examining ASD-related phenotypes, including social interaction, learning, repetitive behaviors, and motor abnormalities. Key ASD-associated molecules such as Neurexin and Neuroligin are conserved in flies, and mutations affecting these proteins impair synapse formation and circuit maturation, leading to behavioral deficits (Bellosta and Soldano, 2019; Tian et al., 2017). *Drosophila* is also a useful system for studying multigenic interactions, as simultaneous manipulation of several genes corresponding to human ASD-associated CNVs results in synergistic worsening of synaptic abnormalities (Grice et al., 2015).

Advanced tools such as GCaMP-based calcium imaging and neuropeptide-release reporters allow direct visualization of circuit activity and synaptic output, enabling the analysis of E/I balance abnormalities in relation to behavior (Streit et al., 2016). In addition, *Drosophila* contains a single *FoxP* gene that corresponds to mammalian *FOXP1/2/4*. *FoxP* contributes to neural development and behavioral regulation, and its loss or knockdown results in impairments in motor coordination, learning, and social behavior (Castells-Nobau et al., 2019; Palazzo et al., 2020). Investigating activity-dependent processes during development and the effects of early-life activity manipulations on mature circuit properties provides valuable insights into developmental vulnerability in ASD (Giachello et al., 2021; Mukherjee and Kanold, 2023).

Presynaptic Ube3a also contributes to circuit maturation, as loss of Ube3a impairs synaptic pruning by failing to degrade presynaptic BMP receptors, leading to abnormal synapse maintenance relevant to Angelman syndrome and ASD (Furusawa et al., 2023).

Insights gained from *Drosophila* studies, including those on synaptic mechanisms, circuit-level regulation, and *FoxP* function, are highly valuable for elucidating ASD-related gene function, E/I balance abnormalities, and multigenic interactions from the molecular to the circuit level. These findings also serve as an important bridge to mammalian models, including mouse systems and human iPSC-derived neurons (Bellosta and Soldano, 2019; Tian et al., 2017; Grice et al., 2015; Castells-Nobau et al., 2019). Furthermore, real-time imaging-based drug screening in flies offers the potential to contribute directly to the development of therapeutic strategies for human ASD (Streit et al., 2016).

## 6 Discussion

*Foxp2* regulates the expression of *VLDLR*, a receptor of Reelin, involved in Wnt signaling pathways essential for the constitution of the cortical layer of humans and mice, as well as Area X development in zebra finches.

Heterozygous mutations in *Foxp2* cause speech-deficits, including ultrasonic vocalizations and singing through cortical and thalamic neuronal networks. whereas homozygous *Foxp2* mutations lead to more severe impairments, affecting thalamocortical and corticothalamic connectivity through the abnormal morphology of brain including cortex, thalamus, and cerebellum.

Future directions include elucidating how *FOXP2* mutations interact with Wnt signaling pathways to regulate cortical and thalamic development and explore their impact on the enteric nervous system and brain–gut axis. Incorporating human brain organoid models will enable validation of these mechanisms in a species-specific context and facilitate functional analysis of language-related circuits. Integrating organoid-based systems with electrophysiology and single-cell transcriptomics could provide critical insights into *FOXP2*-dependent network activity underlying speech and communication. Clinically, *FOXP2*-related disorders and ASD represent promising targets for precision medicine. Potential strategies include gene-editing or RNA-based molecular therapies to restore FOXP2 network integrity and the identification of biomarkers for early ASD diagnosis. These approaches may pave the way for individualized interventions addressing language impairments and broader neurodevelopmental phenotypes, with songbirds (e.g., zebra finches) offering complementary validation of corticostriatal mechanisms for vocal learning.

Despite advances in organoid technology and emerging molecular therapies, animal models are still necessary for analysis on the behavior output. A group of zebrafish models, in particular, will provide complementary data on social interactions and offer insights relevant to human social behavior. *The foxp2*-mutated and *foxp2*-promoter mediated zebrafish models could also be used to specifically test the Wnt/β-catenin hypothesis in the context of *foxp2*-mutated Hirschsprung-like pathology and ENS-related outcomes in future studies because in zebrafish, destruction of the *Lef1/Tcf1* consensus sequence alters the *foxp2* expression in the brain. Recent zebrafish *foxp2* studies show that different genome-editing approaches, such as ZFN and CRISPR/Cas9, yield divergent phenotypes. Because these methods disrupt foxp2 at distinct genomic positions within *foxp2*, a unified understanding of FoxP2 function in zebrafish remains incomplete. Comparative analyses will therefore be important for resolving these discrepancies. Zebrafish also remain a useful model for testing conserved upstream regulation of *foxp2*, including Wnt/β-catenin signaling.

*Drosophila* offers an additional complementary model because it contains a single *FoxP* ortholog (FOXP1/2/4-related) and enables rapid causal testing from gene perturbation to synaptic/circuit phenotypes and ASD-relevant behaviors (locomotion, learning, and social spacing). *Drosophila*, with a single *FoxP* ortholog, offers a non-redundant model enabling rapid causal links from gene perturbation to circuit and behavioral phenotypes.

In the future, integrative analyses that extend from mechanistic studies in *Drosophila* to circuit- and behavior-level validation in zebrafish, songbirds (vocal learning), and mice will be essential for identifying conserved principles of the *FOXP2* network and for pinpointing therapeutic targets.
